# Impact of COVID-19 on the surgical profile of vascular surgery patients at a tertiary hospital in Curitiba, Brazil

**DOI:** 10.1590/1677-5449.202200271

**Published:** 2022-05-23

**Authors:** Giovanna Golin Guarinello, Raissa Campos D’Amico, Ariadne Natalia Mileo Miranda, Jaqueline Novack, Francisco Eduardo Coral

**Affiliations:** 1 Hospital Santa Casa de Curitiba – HSCMC, Curitiba, PR, Brasil.; 2 Universidade Positivo, Curitiba, PR, Brasil..

**Keywords:** coronavirus infections, vascular surgical procedures, amputation

## Abstract

**Background:**

During the COVID-19 pandemic, healthcare services reduced the number of elective procedures performed. Vascular surgery patients are a group at risk of contracting severe forms of the infection, but are also susceptible to complications of their underlying diseases if they do not receive routine care. It is therefore necessary to understand the direct and indirect impacts and consequences of the pandemic on vascular patients.

**Objectives:**

To assess the impact of 1 year of the pandemic on a vascular surgery service and changes to the profile of surgeries during the same period.

**Methods:**

An analysis was conducted of the medical records of patients who underwent elective and emergency surgery from 2019 to 2021. A review of the literature was also conducted, using the search terms “vascular surgery”, “COVID-19”, and “amputations”. Data were analyzed with Stata/SE v.14.1 (StataCorpLP, United States).

**Results:**

A total of 1,043 surgeries were identified during the study period, 51.6% conducted pre-pandemic and 48.4% performed during the pandemic. There was a reduction in the number of elective surgeries and an increase in the number of lower limb amputations and surgical debridements. Increases were also observed in the proportion of patients with peripheral arterial occlusive disease with advanced Rutherford classifications and in the number of cases of diabetic foot.

**Conclusions:**

The reduction in elective care and patients’ reluctance to seek health services during the pandemic are the probable causes of increased severity of patient status, with greater need for lower limb amputation and surgical debridement and changes to the profile of the surgery performed at the service.

## INTRODUCTION

Covid-19, the disease caused by the severe acute respiratory syndrome coronavirus 2 (SARS-CoV-2), was declared a pandemic by the World Health Organization (WHO) on March 11, 2020. The first case in Brazil was confirmed on February 26, 2020, in São Paulo. One month later, cases of the disease had been notified by all of Brazil’s states.^1^ One of the main initial measures taken to combat the disease was social distancing, since there were no vaccines or treatments with proven efficacy. Also on March 11, 2020, starting in the Federal District, these measures began to be implemented in Brazil. In the state of Paraná, measures were implemented in the second week of March.^1^ In order to deal with the elevated number of cases and also as an attempt to contain the disease, significant changes were made to hospital routine and medical consultations all over the world. As seen globally,^2^^-^5 on March 23, 2020, outpatients consultations and elective surgeries provided at our service on the Brazilian National Health Service (SUS - Sistema Único de Saúde) were suspended, leaving patients with emergency and urgent care services as the only care options. In the United States, 80.5% of vascular surgeons started to only operate emergency and urgent cases during the pandemic and this change was regardless of the number of cases observed in each region of the country.^6^

While there has been much discussion of the impacts of COVID-19 on healthcare systems, because of the severity of the disease and bed shortages, in Brazil little has been said about its impacts on diseases unrelated to the virus. The vascular surgery specialty treats a vast range of diseases, including arterial diseases and the diabetic foot. Epidemiologically, patients with these conditions are generally elderly and have multiple comorbidities, placing them in the group of people at risk of contracting severe forms of COVID-19.^7^^-^9 These are vulnerable patients who are susceptible to developing devastating complications of their underlying diseases if their comorbidities are not controlled and they do not receive regular medical attention. Compounding the delays in seeking health services caused by fear of infection by COVID-19, assessment of vascular patients’ need for surgery also became a challenge. It became necessary to weigh the risk of the patient contracting the virus against the risk of the patient developing devastating complications of the underlying vascular diseases.^8^

It is therefore pertinent to assess and discuss the pandemic’s impacts on patients with vascular diseases, in addition to the harm caused by the respiratory disease itself, to enable a rethink of public health policies and find ways to avoid the same problems faced in this pandemic in a possible future situation of a global disease outbreak.

## OBJECTIVE

The objectives of this study were to assess the impact of 1 year of the COVID-19 pandemic on a vascular surgery service at a teaching hospital in Brazil and to evaluate the changes to the profile of surgeries conducted during the same period.

## METHODOLOGY

A retrospective study based on analysis of data from the medical records of patients admitted via the SUS and the private healthcare system who underwent elective, urgent, or emergency surgery from March 2019 to March 2021. Surgeries conducted during the period were analyzed, classifying those performed from March 2019 to March 2020 as pre-pandemic and those from March 2020 to March 2021 as during the pandemic. It should be understood that, throughout the text, whenever references are made to the pandemic period, the reference is only to the study period, since the pandemic is still ongoing at the time of article submission.

The present study was approved by the Research Ethics Committee under protocol number 45138821.7.0000.0099, decision number 4.735.088.

In the statistical analysis, ages are expressed as means, standard deviations, and minimums and maximums. For age, the null hypothesis tested was that mean patient age is equal for the two periods, versus the alternative hypothesis of different means. Categorical variables are expressed as frequencies and percentages. For each of the categorical variables, the null hypothesis tested was that distributions of variable categories are equal during the two periods, versus the alternative hypothesis that distributions are different.

Student’s *t* test for independent samples was used to compare the periods before and during the pandemic in terms of age. Categorical variables were analyzed using Fisher’s exact test or the chi-square test.

P values < 0.05 indicate statistical significance. Data were analyzed with Stata/SE v.14.1 (StataCorpLP, United States).

Finally, a review of the literature was also conducted using the search platforms PubMed, DynaMed, UpToDate, and Google Scholar. Search terms employed included “vascular surgery”, “COVID-19”, and “amputations”.

## RESULTS

A total of 1,043 admissions were identified for the vascular surgery specialty during the period from March 1, 2019 to March 17, 2021, 538 (51.6%) of which occurred before the pandemic (from March 1, 2019 to March 20, 2020) and 505 (48.4%) of which were during the pandemic (March 24, 2020 to March 17, 2021). Some patients were treated in both periods and were included in analyses of both. Patients were assessed for age and main comorbidities during the periods considered pre-pandemic and during the pandemic, as a means of measuring changes to the profile of patients, as specified in Table 1.

**Table 1:** Profile of the patients analyzed during the study.

**Variable**	**Classification**	**Period**		p^*^
		**Pre-pandemic**	**During the pandemic**	
Age (years)		59.9 ± 13.9 (18–92)	62.6 ± 15.0 (19 – 95)	0.007
Sex	Female	265 (57.9%; 4.5%)	182 (46.9%; 5%)	
	Male	193 (42.1%; 4.5%)	206 (53.1%; 5%)	0.001
SAH	No	216 (56.3%; 5%)	134 (37.4%; 5%)	
	Yes	168 (43.8%; 5%)	224 (62.6%; 5%)	<0.001
Additional comorbidities	No	325 (71%; 4.2%)	187 (48.2%; 5%)	
	Yes	133 (29%; 4.2%)	201 (51.8%; 5%)	<0.001
Smoking	No	36 (40.9%; 10.3%)	8 (13.6%; 8.7%)	
	Yes	33 (37.5%; 10.1%)	39 (66.1%; 12.1%)	
	Ex	19 (21.6%; 8.6%)	12 (20.3%; 10.3%)	0.001
DM	No	365 (90.3%; 2.9%)	265 (72.6%; 4.6%)	
	Yes	39 (9.7%; 2.9%)	100 (27.4%; 4.6%)	<0.001
Diagnosis	PAOD	92 (20.1%; 3.7%)	85 (21.9%; 4.1%)	
	Diabetic foot	19 (4.1%; 1.8%)	56 (14.4%; 3.5%)	
	PAOD + diabetic foot	8 (1.7%; 1.2%)	24 (6.2%; 2.4%)	
	Other	339 (74%; 4%)	223 (57.5%; 4.9%)	<0.001

SAH: systemic arterial hypertension; DM: diabetes mellitus; PAOD: peripheral arterial occlusive disease.

* Results expressed as frequency (percentage; 95% margin of error).

Presence of comorbidities was heterogeneous, with greatest prevalence of hypertension (62.6% vs. 43.8%), diabetes (27.4% vs. 9.7%), and other comorbidities, such as dyslipidemia (51.8% vs. 29%), among patients seen during the pandemic and during the pre-pandemic period, respectively (p < 0.05). There was also a robust statistical difference in mean age between the two groups (p = 0.007), with a higher mean age for patients seen during the pandemic.

**Figure 1:**
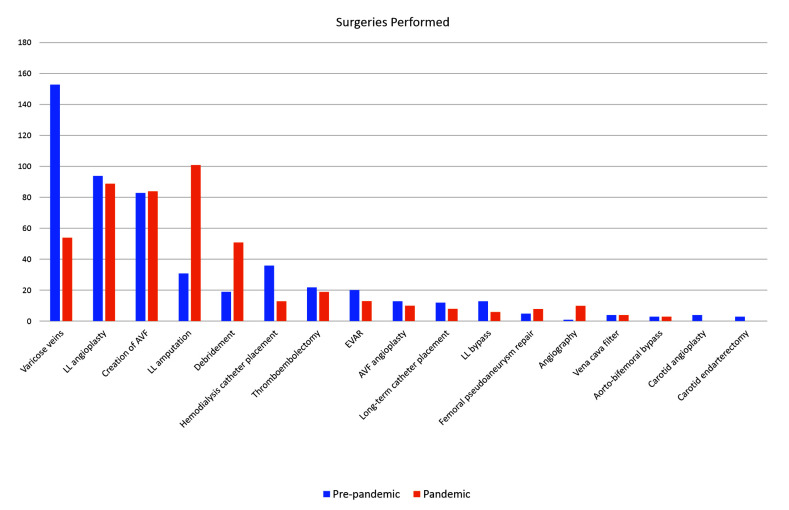
Surgeries performed during the pre-pandemic and pandemic study periods. LL: lower limb; AVF: arteriovenous fistula; EVAR: endovascular abdominal aortic aneurysm repair.

Figure 1 illustrates the change in the profile of surgeries between the two periods analyzed. There was a considerable reduction in the number of elective surgeries, especially surgical treatment of varicose veins (153 pre-pandemic vs. 54 during the pandemic) and there were significant increases in the number of lower limb amputation procedures (31 pre-pandemic vs. 101 during the pandemic) and surgical debridements (19 pre-pandemic vs. 51 during the pandemic).

Table 2 shows a comparison of the two periods in terms of variables related to admission. There was a statistically significant difference (p = 0.001) in the number of admissions of patients with peripheral arterial occlusive disease (PAOD) at Rutherford classes 4, 5, and 6 during the pandemic, when compared with the period before it. There was also a statistical significance (p < 0.001) with relation to the increased number of lower limb amputations, with a predominance of minor amputations, but the difference in the ratio of major to minor amputations was not statistically significant (p = 0.544). There was also evidence of a greater number of patients diagnosed with diabetic foot with complications during the pandemic period (p < 0.001).

**Table 2:** Comparison of admissions during the pre-pandemic and pandemic study periods.

**Variable**	**Classification**	**Pre-pandemic**	**Pandemic**	p^*^
Rutherford	2	4 (3.1%; 3%)	1 (0.6%; 1.1%)	
	3	50 (39.1%; 8.5%)	11 (6.2%; 3.5%)	
	4	24 (18.8%; 6.8%)	43 (24.2%; 6.3%)	
	5	40 (31.3%; 8%)	100 (56.2%; 7.3%)	
	6	10 (7.8%; 4.6%)	23 (12.9%; 4.9%)	<0.001
Amputation	No	503 (93.5%; 2.1%)	393 (77.8%; 3.6%)	
	Yes	35 (6.5%; 2.1%)	112 (22.2%; 3.6%)	<0.001
Amputation classification (restricted to amputation cases)	Minor	25 (71.4%; 15%)	73 (65.2%; 8.8%)	
	Major	10 (28.6%; 15%)	39 (34.8%; 8.8%)	0.544
Diagnosis	PAOD	110 (20.4%; 3,4%)	124 (24.6%; 3.8%)	
	Diabetic foot	27 (5%;1.8%)	83 (16.4%; 3.2%)	
	PAOD + diabetic foot	8 (1.5%; 1%)	38 (7.5%; 2.3%)	
	Others	393 (73%; 3.7%)	260 (51.5%; 4.4%)	<0.001
PAOD	No	420 (78.1%; 3.5%)	343 (67.9%; 4.1%)	
	Yes	118 (21.9%; 3.5%)	162 (32.1%; 4.1%)	<0.001
Diabetic foot (with or without PAOD)	No	503 (93.5%; 2.1%)	384 (76.0%; 3.7%)	
	Yes	35 (6.5%; 2.1%)	121 (24.0%; 3.7%)	<0.001

PAOD: peripheral arterial occlusive disease.

* Fisher’s exact test or chi-square test, p<0.05.

Complications possibly related to COVID-19 were also analyzed for the pandemic period, as shown in Table 3. At our service, just 1.6% of surgeries were performed because of COVID-19. Moreover, just 18 (3.6%) of the 505 patients admitted during the pandemic period were contaminated with SARS-CoV-2 during admission. Mortality among those who were contaminated was 33.3%.

**Table 3:** Complications of COVID-19 in vascular surgery patients.

**Variable**	**Classification**	**n**	%*
**Vascular surgeries due to COVID-19**	No	416	98.3%; 1.2%
	Yes	7	1.6%; 1.2%
**Contamination with COVID-19 during hospital stay**	No	487	96.4%; 1.6%
	Yes	18	3.6%; 1.6%
**Death from COVID-19 or its complications**	No	12	66.7%; 21.8%
	Yes	6	33.3%; 21.8%

* 95% margin of error.

## Discussion

The impacts of social distancing measures and the consequent reduction in healthcare provision have been investigated in several different countries, demonstrating that, since access to medical consultations in general was restricted, compounded by reluctance to seek care at hospitals that also treat COVID-19 cases, fewer patients sought medical attention.^10^ However, those who did attend medical consultations generally had more severe and more advanced diseases, with fewer treatment possibilities and had more complications as a consequence, primarily amputations.^2^^,^^4^^,^^11^

In the present study, important statistically significant changes were observed in the profile of patients during the pandemic. During this period, there was a predominance of male patients and an increase in the proportion of patients admitted with other comorbidities, including systemic arterial hypertension (SAH), diabetes mellitus (DM), and other comorbidities.

In addition to the change in the profile of the patients, an important change was also observed in the types of surgeries conducted during the pandemic period, in particular a sharp drop in the number of varicose veins surgeries. This was expected since these surgeries are elective and elective procedures were prohibited during the pandemic. There was also a considerable increase in the numbers of amputations and debridements. Similar data were reported at other centers, where sharp drops were seen in the number of elective surgeries^7^^,^^10^ and significant increases were observed in the number of amputations.^2^^,^^3^^,^^9^^,^^11^ In a study published by Ilonzo et al.,^12^ for example, there was a 74% reduction in the number of elective surgeries, with an increase only in limb amputations, the frequency of which increased from 15.2% in 2019 to 37.7% during the pandemic period in 2020.

Analyzing the increase in the number of amputations, explanations for this observation include the increased severity with which patients arrived at hospital, primarily because of loss of control of comorbidities and reluctance to seek health services early. One possible cause of the increase may have been the attempt to minimize exposure of patients to COVID-19, avoiding long hospital stays, and also to increase the number of beds available for more severe cases. Given that amputations require a shorter length of hospital stay and need less intensive care unit (ICU) care than lower limb revascularization surgery, amputations may have been considered earlier by the vascular surgeon and even by the patient.^2^^,^^3^ Another way of attempting to minimize prolonged hospital stays and a need for ICU beds at large tertiary centers was to prioritize endovascular treatment over conventional revascularization with bypasses, since it tends to be associated with reduced need for ICU and faster postoperative recovery.^13^ In addition to the overall increase in number of amputations, other articles also report proportional increases in the number of amputations classified as major (those above the ankle), which is an indirect indication of greater severity in these patients,^11^ although this finding did not attain statistical significance in the present study.

When patients with PAOD were analyzed in isolation, it was observed that there was a statistically significant increase in the number of admissions of patients at Rutherford classes 4, 5, and 6, even though the number of revascularization surgery procedures (angioplasty and bypasses) had fallen during the period. A study conducted in Holland also demonstrated an important increase in patients with Rutherford classifications over 5.^11^ The same was reported in Campania, Italy, where, although the absolute number of cases of the disease dropped from 74 cases per 100,000 inhabitants/year to 25 cases per 100,000 inhabitants/year during the lockdown period, the number of cases at Rutherford stages greater than 4 increased.^2^ This finding was associated with an increase in the number of patients admitted with uncontrolled diabetes, which could be another reason for the considerable increase in the number of amputations during the period.

Admissions due to complications of diabetic foot also increased sharply, with statistical significance, during the period analyzed. During the pandemic period, the greater severity of the patients treated increased the likelihood of diabetic patients undergoing amputations to 10.8 times greater than during the pre-pandemic period, with a higher probability of major amputations than minor ones.^14^ A study conducted in India, which has the second largest population of diabetics in the world, assessed the behavior of glycated hemoglobin during periods when access to health services became difficult, such as after earthquakes and tsunamis, in order to estimate the progression and possible complications to be expected in these patients during lockdown. The study estimated a 2.26% increase in glycated hemoglobin for 30 days of isolation and 3.68% for 45 days. In addition to the increase in hemoglobin associated with 30 days of lockdown, it was also estimated that there would be a 10% increase in amputations in 1 year and the percentage would be progressive as the duration of isolation increased.^15^

Many vascular surgery services around the world reported important increases in the occurrence of cases of acute arterial occlusion requiring surgical treatment. In Italy, for example, approximately 60% of cases of acute arterial occlusion occurred in patients positive for COVID-19.^13^ At our service, this increase in the number of patients with acute arterial occlusion was not observed, when compared with the pre-pandemic period, despite our Hospital being a center for referrals of COVID-19 cases and for emergency vascular surgery.^13^

Despite the concern with relation to the possibility of vascular patients contracting the virus and developing severe cases of the disease, just 3.4% of the patients admitted via our service contracted the disease. Over the entire pandemic period analyzed, seven vascular surgeries were performed because of COVID-19, accounting for just 1.6% of the vascular surgeries performed during the pandemic period.

In Singapore, teleconsultations were used as a way to reduce the impact of suspension of consultations and elective surgeries, providing a means for screening patients to determine which patients could be managed with guidance only and which patients required assessment by specialists, in settings with no suspected cases.^3^ This policy could not be adopted at our service because, at that time, there was no provision for teleconsultation with SUS physicians in our region.

Since this study assessed just one pandemic period and only considered patients admitted to a single hospital in the city, the possibility of even more devastating consequences for this specific group of patients cannot be ruled out. The elevated number of amputations will have immeasurable future consequences not only for the individuals involved, but also for healthcare services and expenditure in general. In the future, methods for assessing and providing care to patients of this degree of complexity during periods in which isolation is required will be essential to prevent consequences of lack of access to medical attention that are even more devastating than the disease itself.

## CONCLUSIONS

The reduction in consultations and patients’ reluctance to attend health services during the first year of the pandemic had a negative effect on care for vascular patients. This was the result of a considerable increase in the severity of patients who were admitted and changes to the profile of surgery conducted at the service, with increased numbers of debridements and amputations and reduced numbers of varicose veins surgeries.
