# Supplementary material for: Impact of COVID-19 on the surgical profile of vascular surgery patients at a tertiary hospital in Curitiba, Brazil
Source: J Vasc Bras. 2022 May 23;21:e20220027. doi: 10.1590/1677-5449.202200271 (PMC9136686; doi:10.1590/1677-5449.202200271)
Supplement: Translation

# Translation: Impacto da COVID-19 no perfil cirúrgico dos pacientes de cirurgia vascular em serviço de referência em Curitiba

**DOI:** 10.1590/1677-5449.202200272

**Authors:** Giovanna Golin Guarinello, Raissa Campos D’Amico, Ariadne Natalia Mileo Miranda, Jaqueline Novack, Francisco Eduardo Coral

**Affiliations:** 1 Hospital Santa Casa de Curitiba – HSCMC, Curitiba, PR, Brasil.; 2 Universidade Positivo, Curitiba, PR, Brasil.

**Keywords:** infecções por coronavírus, procedimentos cirúrgicos vasculares, amputação

## Abstract

**Contexto:**

Na pandemia de covid-19, os serviços de saúde diminuíram os atendimentos e procedimentos eletivos. Pacientes de cirurgia vascular são grupo de risco para adquirir formas graves da infecção, ao mesmo tempo que são suscetíveis a apresentar complicações de suas doenças de base caso não tenham acompanhamento rotineiro. Dessa forma, faz-se necessário entender os impactos e as consequências diretas e indiretas da pandemia com relação aos pacientes vasculares.

**Objetivos:**

Avaliar o impacto de 1 ano de pandemia em um serviço de Cirurgia Vascular, assim como a mudança do perfil de cirurgias no mesmo período.

**Métodos:**

Foi feita a análise de prontuários de pacientes submetidos a cirurgias eletivas e de urgência entre 2019 e 2021. Em conjunto, foi realizada uma revisão de literatura com as palavras-chave “cirurgia vascular”, “covid-19” e “amputações”. Os dados foram analisados com o programa computacional Stata/SE v.14.1 (StataCorpLP, EUA).

**Resultados:**

Foram identificadas 1.043 cirurgias no período de estudo, sendo 51,6% pré-pandemia e 48,4% durante a pandemia. Observou-se redução no número de cirurgias eletivas e aumento no número de amputações de membros inferiores e desbridamentos cirúrgicos. Foi possível observar também aumento de pacientes com doença arterial obstrutiva periférica com classificação de Rutherford avançada, assim como de casos de pé diabético.

**Conclusões:**

A diminuição dos atendimentos eletivos e o receio dos pacientes em procurar os serviços de saúde durante o período da pandemia são os prováveis motivos que justificam o aumento da gravidade dos quadros dos pacientes, com maior necessidade de amputação de membros inferiores, desbridamento cirúrgico e mudanças no perfil de cirurgia do serviço.

## INTRODUÇÃO

A covid-19, doença causada pelo coronavírus 2 da síndrome respiratória aguda grave (SARS-CoV-2), foi declarada como pandemia pela Organização Mundial de Saúde (OMS) no dia 11 de março de 2020. O primeiro caso no Brasil foi confirmado em 26 de fevereiro de 2020, na cidade de São Paulo. Após 1 mês, todos os estados brasileiros já haviam notificado casos da doença^1^. Uma das principais medidas iniciais para o combate da doença foi o distanciamento social, uma vez que, até então, não havia vacinas ou tratamentos efetivos comprovados. Também em 11 de março de 2020, inicialmente no Distrito Federal, essas medidas começaram a ser implantadas no Brasil. No Paraná, o mesmo ocorreu na segunda semana de março^1^. Como forma de atender o número elevado de casos e como uma tentativa de conter a doença, uma importante mudança na rotina de hospitais e atendimentos médicos se instalou em todo o mundo. Como observado mundo afora^2^^-^5, em nosso serviço, no dia 23 de março de 2020, os atendimentos ambulatoriais, assim como as cirurgias eletivas do Sistema Único de Saúde (SUS), foram suspensos, deixando aos pacientes apenas as opções de buscas em serviços de pronto atendimento. Nos Estados Unidos, 80,5% dos cirurgiões vasculares passaram a operar apenas casos de urgência e emergência durante a pandemia, sendo essa mudança observada independentemente do número de casos observados na região do país^6^.

Apesar de muito se discutir sobre os impactos da covid-19 nos sistemas de saúde, devido à gravidade da doença e à falta de leitos, pouco se fala, no Brasil, sobre os seus impactos em doenças não relacionadas com o vírus. A especialidade de cirurgia vascular inclui tratamento de uma vasta variedade de doenças, entre elas, as doenças arteriais e o pé diabético. Epidemiologicamente, os portadores dessas enfermidades geralmente são idosos, que apresentam múltiplas comorbidades, o que os coloca no grupo de risco para adquirir as formas graves da covid-19^7^^-^9. São pacientes vulneráveis e que estão suscetíveis a apresentar complicações devastadoras de suas doenças de base caso não tenham suas comorbidades controladas e um acompanhamento médico periódico. Dessa forma, além da demora na procura pelos serviços de saúde por receio de adquirir a infecção pela covid-19, a avaliação da necessidade de cirurgias dos pacientes vasculares se tornou também um desafio. Foi necessário pesar o risco de o paciente adquirir o vírus *versus* o risco de o paciente evoluir com complicações devastadoras de suas doenças vasculares de base^8^.

Sendo assim, são válidas a avaliação e a discussão dos impactos que a pandemia trouxe para os pacientes portadores de doenças vasculares, além dos malefícios causados pela própria doença respiratória, para que seja possível repensar as políticas de saúde pública e encontrar formas de evitar os mesmos problemas enfrentados nesta pandemia em uma possível nova situação de doença de acometimento mundial.

## OBJETIVO

O objetivo deste estudo foi avaliar o impacto de 1 ano de pandemia de covid-19 em um serviço de Cirurgia Vascular de um hospital-escola no Brasil, assim como avaliar a mudança do perfil de cirurgias durante o mesmo período.

## METODOLOGIA

Estudo retrospectivo, realizado a partir da análise de dados de prontuários de pacientes internados, do SUS e do sistema privado, submetidos a cirurgias eletivas, de urgência e emergência, no período de março de 2019 a março de 2021. Foram avaliadas as cirurgias realizadas no período, sendo que as cirurgias entre março de 2019 e março de 2020 foram consideradas pré-pandemia, e as cirurgias entre março de 2020 e março de 2021 foram consideradas como pandemia. Entende-se que, durante o texto, sempre que nos referirmos ao período de pandemia, este consiste apenas no período do estudo, uma vez que a pandemia ainda está em vigência na data da submissão deste artigo.

O presente estudo foi aprovado pelo Comitê de Ética em Pesquisa sob o número 45138821.7.0000.0099, número do parecer 4.735.088.

Na análise estatística, as descrições da idade foram apresentadas por meio de média, desvio padrão, mínimo e máximo. Para idade, testou-se a hipótese nula de que as médias dos pacientes são iguais nos dois períodos *versus* a hipótese alternativa de médias diferentes. Variáveis categóricas foram descritas por frequência e percentual. Para cada uma das variáveis categóricas, testou-se a hipótese nula de que as distribuições sobre as classificações da variável são iguais para os dois períodos *versus* a hipótese alternativa de distribuições diferentes.

Para a comparação dos períodos antes e durante a pandemia, em relação à idade, foi usado o teste *t* de Student para amostras independentes. Variáveis categóricas foram analisadas, usando-se o teste exato de Fisher ou o teste de qui-quadrado.

Valores de p < 0,05 indicaram significância estatística. Os dados foram analisados com o programa computacional Stata/SE v.14.1 (StataCorpLP, EUA).

Por fim, em conjunto, foi realizada uma revisão de literatura com base nas plataformas de pesquisa PubMed, DynaMed, UpToDate e Google Scholar. Os temas pesquisados envolveram as palavras-chave “cirurgia vascular”, “covid-19” e “amputações”.

## RESULTADOS

Foram identificados 1.043 internamentos para a especialidade de cirurgia vascular no período de 01/03/2019 a 17/03/2021, sendo 538 (51,6%) antes da pandemia (de 01/03/19 a 20/03/20) e 505 (48,4%) durante a pandemia (24/03/20 a 17/03/21). Alguns pacientes tiveram atendimento nos dois períodos e foram incluídos em ambos. Os pacientes foram avaliados quanto à idade e às principais comorbidades no período considerado pré e durante a pandemia, como forma de mensurar mudanças no perfil dos pacientes conforme especificado na Tabela 1.

**Tabela 1:** Perfil de pacientes analisados durante o estudo.

**Variável**	**Classificação**	**Período**		p*
		**Pré-pandemia**	**Durante a pandemia**	
Idade (anos)		59,9 ± 13,9 (18-92)	62,6 ± 15,0 (19-95)	0,007
Sexo	Feminino	265 (57,9%; 4,5%)	182 (46,9%; 5%)	
	Masculino	193 (42,1%; 4,5%)	206 (53,1%; 5%)	0,001
HAS	Não	216 (56,3%; 5%)	134 (37,4%; 5%)	
	Sim	168 (43,8%; 5%)	224 (62,6%; 5%)	<0,001
Tem outras comorbidades	Não	325 (71%; 4,2%)	187 (48,2%; 5%)	
	Sim	133 (29%; 4,2%)	201 (51,8%; 5%)	<0,001
Tabagismo	Não	36 (40,9%; 10,3%)	8 (13,6%; 8,7%)	
	Sim	33 (37,5%; 10,1%)	39 (66,1%; 12,1%)	
	Ex	19 (21,6%; 8,6%)	12 (20,3%; 10,3%)	0,001
DM	Não	365 (90,3%; 2,9%)	265 (72,6%; 4,6%)	
	Sim	39 (9,7%; 2,9%)	100 (27,4%; 4,6%)	<0,001
Diagnóstico	DAOP	92 (20,1%; 3,7%)	85 (21,9%; 4,1%)	
	Pé diabético	19 (4,1%; 1,8%)	56 (14,4%; 3,5%)	
	DAOP + pé diabético	8 (1,7%; 1,2%)	24 (6,2%; 2,4%)	
	Outro	339 (74%; 4%)	223 (57,5%; 4,9%)	<0,001

HAS: hipertensão arterial sistêmica; DM: diabetes melito; DAOP: doença arterial obstrutiva crônica.

* Resultados apresentados por frequência (percentual; margem de erro de 95%).

A presença de comorbidades foi bastante heterogênea, com maior prevalência de hipertensão (62,6% vs. 43,8%), diabetes (27,4% vs. 9,7%) e outras comorbidades, como dislipidemia (51,8% vs. 29%), nos pacientes atendidos durante a pandemia, quando comparados aos pacientes atendidos no período pré-pandemia (p < 0,05). Com relação à média de idade, também foi observada uma importante diferença estatística entre os dois grupos (p = 0,007), com a maior média de idade observada em pacientes atendidos durante a pandemia.

Na Figura 1, observa-se a mudança do padrão das cirurgias realizadas quando comparados os períodos analisados. Há uma redução importante no número de cirurgias eletivas, especialmente o tratamento cirúrgico de varizes (153 pré-pandemia vs. 54 durante a pandemia), bem como nota-se um aumento importante no número de cirurgias de amputação de membro inferior (31 pré-pandemia vs. 101 durante a pandemia) e desbridamentos cirúrgicos (19 pré-pandemia vs. 51 durante a pandemia).

**Figura 1:** Cirurgias realizadas pré-pandemia vs. período de estudo do trabalho durante a pandemia. MI: membro inferior; FAV: fístula arteriovenosa; EVAR: terapia endovascular do aneurisma da aorta abdominal.

Na Tabela 2, observa-se uma comparação dos períodos quanto a variáveis relativas ao internamento. Há diferença estatística significativa (p = 0,001) no número de internamentos de pacientes portadores de doença arterial obstrutiva periférica (DAOP) nas classes 4,5 e 6 de Rutherford, durante a pandemia, quando comparado ao período antes disso. Ainda, nota-se significância estatística (p < 0,001) com relação ao aumento do número de amputações de membros inferiores, com predominância das amputações menores, mas sem diferença estatística quando comparadas amputações maiores *versus* menores (p = 0,544). Há, também, evidência de maior número de pacientes diagnosticados com pé diabético, com complicações, no período da pandemia (p < 0,001).

**Tabela 2:** Comparação pré-pandemia e período de estudo do trabalho na pandemia quanto ao internamento.

**Variável**	**Classificação**	**Pré-pandemia**	**Pandemia**	p*
Rutherford	2	4 (3,1%; 3%)	1 (0,6%; 1,1%)	
	3	50 (39,1%; 8,5%)	11 (6,2%; 3,5%)	
	4	24 (18,8%; 6,8%)	43 (24,2%; 6,3%)	
	5	40 (31,3%; 8%)	100 (56,2%; 7,3%)	
	6	10 (7,8%; 4,6%)	23 (12,9%; 4,9%)	<0,001
Amputação	Não	503 (93,5%; 2,1%)	393 (77,8%; 3,6%)	
	Sim	35 (6,5%; 2,1%)	112 (22,2%; 3,6%)	<0,001
Classificação da amputação (restrito a casos de amputação)	Menor	25 (71,4%; 15%)	73 (65,2%; 8,8%)	
	Maior	10 (28,6%; 15%)	39 (34,8%; 8,8%)	0,544
Diagnóstico	DAOP	110 (20,4%; 3.4%)	124 (24,6%; 3,8%)	
	Pé diabético	27 (5%;1,8%)	83 (16,4%; 3,2%)	
	DAOP + pé diabético	8 (1,5%; 1%)	38 (7,5%; 2,3%)	
	Outro	393 (73%; 3,7%)	260 (51,5%; 4,4%)	<0,001
DAOP	Não	420 (78,1%; 3,5%)	343 (67,9%; 4,1%)	
	Sim	118 (21,9%; 3,5%)	162 (32,1%; 4,1%)	<0,001
Pé diabético (com ou sem DAOP)	Não	503 (93,5%; 2,1%)	384 (76,0%; 3,7%)	
	Sim	35 (6,5%; 2,1%)	121 (24,0%; 3,7%)	<0,001

DAOP: doença arterial obstrutiva crônica.

* Teste exato de Fisher ou teste de qui-quadrado, p<0,05.

Foram avaliadas, também, possíveis complicações relacionadas com a covid-19 durante o período da pandemia, como demonstrado na Tabela 3. Em nosso serviço, apenas 1,6% das cirurgias foram realizadas em decorrência da covid-19. Ademais, dos 505 pacientes internados durante a pandemia, apenas 18 (3,6%) foram contaminados pelo SARS-CoV-2 durante o internamento. Dos contaminados, a mortalidade foi de 33,3%.

**Tabela 3:** Complicações da covid-19 nos pacientes de cirurgia vascular.

**Variável**	**Classificação**	**n**	%*
**Cirurgias vasculares devido à covid-19**	Não	416	98,3%; 1,2%
	Sim	7	1,6%; 1,2%
**Contaminação com covid-19 durante internamento**	Não	487	96,4%; 1,6%
	Sim	18	3,6%; 1,6%
**Morte em decorrência da covid-19 ou de suas complicações**	Não	12	66,7%; 21,8%
	Sim	6	33,3%; 21,8%

* Margem de erro 95%.

## DISCUSSÃO

Os impactos das medidas de distanciamento social e, consequentemente, da diminuição dos atendimentos em saúde foram avaliados em diversos países, demonstrando que, uma vez que os acessos aos atendimentos médicos em geral estavam restritos, em conjunto com o receio de buscar atendimento em hospitais que também atendiam casos de covid-19, menos pacientes procuraram atendimento médico^10^. No entanto, aqueles que procuravam consulta médica geralmente apresentavam doenças mais graves e avançadas, com menor possibilidade de tratamento e consequente aumento de suas complicações, principalmente amputações^2^^,^^4^^,^^11^.

No presente estudo, foi possível observar, com significância estatística, uma importante mudança de perfil dos pacientes durante a pandemia. Durante esse período, foi observada predominância de pacientes do sexo masculino, assim como aumento de pacientes internados que possuíam outras comorbidades, entre elas hipertensão arterial sistêmica (HAS), diabetes melitos (DM) e outras comorbidades.

Além de alteração no perfil dos pacientes, foi possível observar também uma importante mudança nos tipos de cirurgias realizadas no período da pandemia, com destaque para importante diminuição de cirurgias de varizes. Isso era esperado, dado o fato de ser uma cirurgia de cunho eletivo, cuja realização foi proibida durante a pandemia. Notou-se, também, um importante aumento do número de amputações e desbridamentos. Tais dados igualmente foram observados em outros centros, onde foi possível verificar importante queda do número de cirurgias eletivas^7^^,^^10^ e aumento do número de amputações^2^^,^^3^^,^^9^^,^^11^. No estudo publicado por Ilonzo et al.^12^, por exemplo, houve uma redução de 74% no número de cirurgias eletivas, com aumento na realização apenas de amputações de membros, cuja frequência, que em 2019 era de 15,2%, saltou para 37,7% durante o período da pandemia em 2020.

Quando analisamos o aumento do número de amputações, algumas justificativas encontradas para tal fato são a maior gravidade com que os pacientes chegaram ao hospital, principalmente pelo descontrole de comorbidades e pelo receio de procurar os serviços de saúde precocemente. Outra possível causa para o aumento pode ter sido a tentativa de minimizar a exposição dos pacientes à covid-19, evitando internamentos prolongados, bem como visando aumentar o número de leitos para pacientes de maior gravidade. Dado que as amputações possuem menor tempo de internamento e menor necessidade de unidade de terapia intensiva (UTI) do que cirurgias de revascularização de membros inferiores, as amputações podem ter sido consideradas de forma mais precoce pelo médico cirurgião vascular e até mesmo pelo paciente^2^^,^^3^. Outra forma de tentar minimizar o internamento prolongado e a necessidade de vagas de UTI nos grandes centros de referência foi priorizar o tratamento endovascular, e não a revascularização convencional com pontes; afinal, o primeiro procedimento tende a estar associado a menor necessidade de UTI e também a uma recuperação pós-operatória mais rápida^13^. Além do aumento geral do número de amputações, outros artigos relataram aumento proporcional do número de amputações consideradas maiores (aquelas realizadas acima do tornozelo), indicando, indiretamente, a maior gravidade desses pacientes^11^, apesar de esse achado não ter significância estatística no presente estudo.

Quando avaliados os pacientes com DAOP isoladamente, foi possível observar, com significância estatística, um aumento no número de internações de pacientes com classes 4,5 e 6 de Rutherford, apesar de o número de cirurgias de revascularização (angioplastia e pontes) ter diminuído no período. Um estudo conduzido na Holanda também demonstrou importante aumento de pacientes com classificações de Rutherford maior que 5^11^. O mesmo foi relatado em Campania Italia, onde, apesar do número absoluto de casos pela doença ter caído de 74 casos por 100.000 habitantes/ano para 25 casos por 100.000 habitantes/ano no período de *lockdown*, o número daqueles com Rutherford estágio maior que 4 aumentou^2^. Tal dado, associado com o aumento do número de pacientes internados por pé diabético descompensado, pode ser mais uma das causas relacionadas com o aumento importante do número de amputações no período.

Os internamentos por complicações de pé diabético também tiveram expressiva mudança, com significância estatística, durante o período avaliado. Durante o período da pandemia, devido à maior gravidade dos pacientes, as chances de pacientes diabéticos evoluírem para amputações foi 10,8 vezes maior do que no período pré-pandemia, com ainda maior probabilidade de evoluir para amputações maiores do que menores^14^. Um estudo conduzido na Índia, onde se concentra a segunda maior população de diabéticos do mundo, avaliou o comportamento da hemoglobina glicada durante períodos de dificuldade de acesso a serviços de saúde, como terremotos e tsunamis, para estimar a evolução e possíveis complicações esperadas para esses pacientes durante o período de *lockdown*, estimando um aumento da hemoglobina glicada de 2,26% para 30 dias e 3,68% para 45 dias de isolamento. Além do aumento da hemoglobina com *lockdown* de 30 dias, foi estimado um aumento de 10% nos casos de amputação em 1 ano, sendo essa porcentagem progressiva quanto maior o tempo de isolamento^15^.

Muitos serviços de cirurgia vascular ao redor do mundo reportaram um aumento importante na ocorrência de casos de oclusão arterial aguda com necessidade de tratamento cirúrgico. Na Itália, por exemplo, aproximadamente 60% dos casos de oclusão arterial aguda ocorreram em pacientes positivos para covid-19^13^. No nosso serviço, não foi observado esse aumento do número de atendimentos por oclusão arterial aguda, quando comparado ao período pré-pandemia, apesar de o nosso Hospital ter sido referência para casos de covid-19 e também para atendimentos em cirurgia vascular emergencial^13^.

Apesar de todo o receio quanto à possibilidade de os pacientes vasculares adquirirem o vírus e evoluírem para doenças graves, apenas 3,4% dos pacientes internados no nosso serviço contraíram a doença. Durante todo o período de pandemia avaliado, foram realizadas sete cirurgias vasculares devido à covid-19, representando apenas 1,6% das cirurgias vasculares realizadas na pandemia.

Em Singapura, como uma forma de minimizar os impactos da suspensão dos atendimentos e cirurgias eletivas, a realização de teleconsultas funcionou como uma forma de triagem daqueles que poderiam ser acompanhados apenas com orientações e daqueles que necessitavam ser avaliados por especialistas, fora do ambiente dos casos suspeitos^3^. O mesmo não pôde ser realizado no nosso serviço, uma vez que, até então, não havia teleconsulta com médicos pelo SUS em nossa região.

Como este estudo avaliou apenas um período da pandemia e considerou apenas os pacientes internados em um único hospital da cidade, não é possível descartar consequências ainda mais devastadoras para esse grupo de pacientes especificamente. O número elevado de amputações trará futuramente consequências imensuráveis não só para esses indivíduos, mas também para os serviços e custos de saúde em geral. No futuro, formas de atendimento e assistência à saúde para pacientes dessa complexidade, em períodos de necessidade de isolamento, serão essenciais para que as consequências da falta de acesso ao atendimento médico não sejam ainda mais devastadoras do que a própria doença.

## CONCLUSÃO

A diminuição dos atendimentos e o receio dos pacientes em procurar os serviços de saúde durante o período de 1 ano de pandemia promoveram efeito negativo no cuidado do paciente vascular. Isso aconteceu porque houve aumento considerável da gravidade dos pacientes internados, assim como mudança no perfil cirúrgico do serviço, o que evidenciou aumento do número de desbridamentos e amputações, além de diminuição das cirurgias de varizes.
